# Natural Regeneration of Trees in Three Types of Afforested Stands in the Taihang Mountains, China

**DOI:** 10.1371/journal.pone.0108744

**Published:** 2014-09-30

**Authors:** Xitian Yang, Dongfeng Yan, Canran Liu

**Affiliations:** 1 College of Forestry, Henan Agricultural University, Zhengzhou, Henan, China; 2 Arthur Rylah Institute for Environmental Research, Department of Environment and Primary Industries, Heidelberg, Victoria, Australia; University of New England, Australia

## Abstract

Natural regeneration is the natural process by which plants replace themselves. It is a cost-effective way to re-establish vegetation, and it helps to preserve genetic identity and diversity. In this study, we investigated the natural regeneration of trees in three types of afforested stands in the Taihang Mountains, China, which were dominated by *Robinia pseudoacacia* (black locust), *Quercus variabilis* (Chinese cork oak) and *Platycladus orientalis* (Chinese arborvitae) respectively. A consistent pattern was found among the three types of stands, being that the density of seedlings was positively correlated with the overstory canopy cover and negatively correlated with the covers of shrub, herb and litter layers. While a positive correlation between the density of seedlings and stand age was found for the conifer stands, negative correlations were found for the two types of broadleaf stands. Correlations between the density of saplings and the stand attributes were not consistent among the three types of stands. The two types of broadleaf stands had higher densities of seedlings and saplings than the conifer stands. While the broadleaf stands had adequate recruits for regeneration, the conifer stands did not have enough recruits. Our findings suggest that the overstory canopy should be prevented from being disturbed, any reduction of the canopy cover will decrease the recruits and affect the regeneration.

## Introduction

Forest natural regeneration is a natural biological process of forest resource reproduction in ecosystem dynamics [1]. During this process tree-dominated plant communities develop and evolve, which has far-reaching impact on the structure of forests in the future [2]. Forest environment can be restored with natural regeneration through seedling establishment and resprouting from cut stumps, which results in high-quality forests with high biodiversity [1]. For the restoration to be successful, it is important to enhance the natural regeneration of trees and create self-sustaining communities [3]. The improvement of natural regeneration in stands may also be the most cost-effective way to obtain a species-rich productive stand [4]. Therefore, understanding the natural regeneration processes and dynamics is crucial to planning and carrying out management activities [5], [6].

The Taihang Mountains in northern China was largely covered by forests in the history. With the intermittent occurring of wars and increase of population the forests were severely destroyed. By the mid of 20^th^ century the forest cover had reduced to less than 5% in this region. Many areas became shrubland even grassland or cropland. Consequently natural disasters occurred more frequently in this region and nearby areas than ever before. In order to protect the environment of these ecologically fragile areas, intensive afforestation has been carried out in this region since 1950 [7]. In 1984 Chinese government initiated a major project, The Taihang Mountains Afforestation Project. It was trialled from 1987 to 1993 and formally started in 1994 (http://etree.forestry.gov.cn/portal/201211/14/c832.html). It was planned to plant 3.56 million ha of forests by 2050 in which 110 counties in this region are involved [8]. By the end of 2011 0.9 million ha had been planted (http://etree.forestry.gov.cn/portal/201211/14/c832.html).

The restored ecosystems in this region have been studied by researchers from different aspects. These include the numbers and biomass of soil microbes and soil respiration rate [9], carbon stock [10], litterfall and litter decomposition dynamics [11]–[13], nutrient dynamics [14]–[16], composition and diversity of soil seed bank [17], [18], and composition, structure and species diversity of plant communities [14], [18], [19]. But to our knowledge, there have been no studies specifically focused on natural regeneration of the afforested stands in this region. Due to its importance in the success of the afforestation, there is a need to carry out a study on this. In this paper, we focus on three types of the planted forests with different ages in the southern part of the Taihang Mountains, and ask these questions: What are the current status of the natural regeneration in terms of seedlings and saplings in these forests? What kind of roles do the forest attributes play in the regeneration?

## Methods

### Study area

The study area is located in the hilly areas near Jiyuan City, Henan Province, China, which is in the southern part of the Taihang Mountains. This is an area with the most severe soil and water erosions in the Yellow River reach. Ecosystems are significantly degraded in some parts of the area. The relatively low precipitation and severe soil condition make it hard to restore vegetation.

It has a warm temperate continental monsoonal climate. The average temperature is 13.1°C, 0.5°C and 26.2°C for annual, January and July respectively. It has averagely 235 days without frost and 3600–4000°C annual cumulative temperature. The annual precipitation is 600–800 mm, and the maximum and minimum annual precipitation is 1060 mm and 360 mm respectively. There is a large variation in rainfall among seasons with much more in summer and autumn than in spring and winter. The rainfall in July, August and September accounts for 60% of the annual total. It is easy to get locally flooded in autumn, which makes soil erosion.

Mountain cinnamonic soils developed from granite-gneiss prevail in this area. Most of the soils are skeletal and not well-structured, and also have large content of gravels, which makes them vulnerable to erosion. The soils are shallow and have limited water retention ability.

The study area belongs to the warm temperate deciduous broadleaved forest district. Since the altitude is locally not very different, the vegetation distribution is based on the differential water and temperature caused by the soil condition and slope aspect. According to the classification system of ‘Vegetation of China’, there are five main types of vegetation in this region, including evergreen coniferous forest, deciduous broadleaved forest, deciduous broadleaved shrubland, shrubs and grasses mixture, and grassland. However, because of severe human disturbance, the main vegetation types in this area have become secondary dry shrubs and grasses mixture. Due to the severe environmental condition, grasslands account for the largest proportion of the study area. The species are mainly drought tolerant, and the vegetation is low in both height and coverage.

### Plot selection

Three types of forests were sampled in the state-owned forests of Jiyuan City (See Table S1 for the specific locations), for which specific permission was not required and in which endangered or protected species were not involved. They had *Robinia pseudoacacia* (black locust), *Quercus variabilis* (Chinese cork oak) and *Platycladus orientalis* (Chinese arborvitae) respectively as the target species at the beginning of planting. Direct seeding was used for the two broadleaved species and seedling planting for the coniferous species. With the help of topography, forest and vegetation maps and the information obtained from pilot survey, 33 plots (20 m×20 m) were selected in the typical stands at three age groups for each of the three types of forests. The three age groups include those stands with age 10–20 for Group 1, 30–40 for Group 2 and 50–55 for Group 3. For each age group, the three types of plots were selected in similar environmental conditions in terms of altitude, aspect, slope position and slope degree. Three or more plots were selected for each combination of type and age group. Any single plot did not cross a river, a road and a hill ridge, and also was far away from forest edges and was within the area with little human disturbance after the afforestation took place.

### Survey

In each plot, we recorded all the individual trees (with diameter at breast height DBH>4.5 cm for broadleaved trees and 0.5 cm for coniferous trees) in terms of species name, DBH, height, crown width, growth status, vigour and layer. We also recorded the overall tree cover of each plot. Canopy cover was measured with crown analyser.

In each plot, 25 2 m×2 m quadrats were randomly located for regeneration survey in terms of seedlings (height≤0.5 m), small saplings (0.5 m<height ≤2 m) and large saplings (height>2 m and diameter ≤4.5 cm for broadleaved trees and 0.5 cm for coniferous trees). Regeneration was characterised with density and frequency. While density is the number of recruits (seedlings and/or saplings according to the context) in a unit area calculated from the 25 quadrats for each plot, frequency is the proportion of quadrats in which recruits occur.

### Data analysis

Kendall's correlation coefficient was used to characterise the relationships of seedling density and sapling density with forest stand attributes. We also compared the density and frequency of the seedlings and saplings between different types of stands, different age groups for each type as well as different combinations of types and age groups. Because of the small sample size for each situation, all the statistical tests were done with randomization method. Therefore, data transformation was not necessary. A 20-year old and a 35-year old black locust stands were excluded from the analyses since they have large numbers of small seedlings, which are more than three (3.16) and four (4.04) times the standard deviation above the average when calculated with the original data and more than twice (2.15 and 2.32) the standard deviation above the average when calculated with the transformed data using ln(x+1) (a positive number, here 1, was added to the logarithmic function because one plot had no seedling at all).

## Results

### Density and frequency of recruits in three types of forests

The densities of seedlings, small saplings and large saplings respectively were 1957, 871 and 143 ha^−1^ for black locust stands, 2192, 1358 and 442 ha^−1^ for oak stands, and 725, 958 and 117 ha^−1^ for conifer stands (Table 1). Both the black locust stands and the oak stands had significantly more seedlings than the conifer stands (p = 0.052 and 0.005 respectively), the oak stands had marginally more saplings than the black locust stands (p = 0.098 and 0.024 for small and large saplings respectively), and the oak stands had significantly more large saplings than the conifer stands (p = 0.004). Generally in most cases there was not much difference in the density of recruits between different age groups for each type of stands (Table 2). Significant differences existed in the seedling densities between different age groups of conifer stands, where age groups 2 and 3 had more seedlings than age group 1, and also in the large sapling densities between different age groups of the oak stands, where density increased from group 1 through group 2 to group 3.

**Table 1:** Density (individual ha^−1^) of recruits for the three stand types and three age groups.

Stand type	Age group	Species planted			Species not planted			All species		
		Seedling	Small sapling	Large sapling	Seedling	Small sapling	Large sapling	Seedling	Small sapling	Large sapling
*R. pseudoacacia*	1	450	500	150	450	400	100	900	900	250
	2	333	33	167	2300	800	0	2633	833	167
	3	150	400	0	1850	500	0	2000	900	0
	overall	314	271	114	1643	600	29	1957	871	143
*Q. variabilis*	1	1925	800	100	400	325	100	2325	1125	200
	2	2150	1275	325	1050	525	75	3200	1800	400
	3	650	925	625	400	225	100	1050	1150	725
	overall	1575	1000	350	617	358	92	2192	1358	442
*P. orientalis*	1	200	233	0	250	467	50	450	700	50
	2	167	0	33	900	967	200	1067	967	233
	3	167	67	0	767	1400	133	933	1467	133
	overall	183	133	8	542	825	108	725	958	117

**Table 2:** Randomization test for the difference in the density (individual ha^−1^) of recruits between different stand types and different age groups.

G1	G2	n1	n2	Seedling			Small sapling			Large sapling		
				Difference		p_v	Difference		p_v	Difference		p_v
				Obs.	Exp.		Obs.	Exp.		Obs.	Exp.	
Between different types for all ages of stands												
RP	QV	7	12	−235	−99	0.435	−487	−12	0.098	−299	18	0.007
RP	PO	7	12	1232	−80	0.052	−87	−19	0.392	26	4	0.432
QV	PO	12	12	1467	−17	0.005	400	17	0.133	325	−8	0.006
Between different stand age groups for *R. pseudoacacia* stands												
AG1	AG2	2	3	−1733	−1733	0.672	67	−17	0.489	83	83	0.503
AG1	AG3	2	2	−1100	1100	0.179	0	0	0.658	250	250	0.511
AG2	AG3	3	2	633	217	0.483	−67	−67	0.694	167	0	0.303
Between different stand age groups for *Q. variabilis* stands												
AG1	AG2	4	4	−875	125	0.293	−675	25	0.138	−200	0	0.060
AG1	AG3	4	4	1275	175	0.170	−25	25	0.439	−525	25	0.009
AG2	AG3	4	4	2150	50	0.128	650	−200	0.232	−325	25	0.032
Between different stand age groups for *P. orientalis* stands												
AG1	AG2	6	3	−617	33	0.062	−267	33	0.236	−183	67	0.085
AG1	AG3	6	3	−483	−33	0.050	−767	33	0.087	−83	17	0.156
AG2	AG3	3	3	133	0	0.432	−500	−33	0.171	100	−33	0.328

G_1: Group 1; G_2: Group 2; n1 and n2: number of plots for Group 1 and Group 2 respectively; AG: age group; RP: *R. pseudoacacia*; QV: *Q. variabilis*; PO: *P. orientalis*; p_v: p_value.

The frequencies of the recruits were low for all the three types of stands, and their average frequencies were all less than 30% (Table 3). The oak stands had higher frequencies of recruits than the other two types (p<0.05 except for black locust small saplings, Table 4). For all the three types of stands, the middle age group had higher frequencies of seedlings and small saplings than the other two age groups.

**Table 3:** Frequency (%) of recruits for the three stand types and three age groups.

Stand type	Age group	Species planted			Species not planted			All species		
		Seedling	Small sapling	Large sapling	Seedling	Small sapling	Large sapling	Seedling	Small sapling	Large sapling
*R. pseudoacacia*	1	18	12	6	16	10	4	26	18	8
	2	13	1	7	21	24	0	33	25	7
	3	4	10	0	20	14	0	22	18	0
	overall	12	7	5	19	17	1	28	21	5
*Q. variabilis*	1	28	18	3	14	10	4	36	26	7
	2	31	28	10	12	9	2	37	34	12
	3	12	20	15	5	6	4	15	24	18
	overall	24	22	9	10	8	3	29	28	12
*P. orientalis*	1	6	8	0	7	11	1	13	18	1
	2	3	0	1	24	23	7	25	23	8
	3	1	1	0	17	29	1	17	29	1
	overall	4	4	0	14	18	3	17	22	3

**Table 4:** Randomization test for the difference in the frequency (%) of recruits between different stand types and different age groups.

G_1	G_2	n1	n2	Seedling			Small sapling			Large sapling		
				Difference		p_v	Difference		p_v	Difference		p_v
				Obs.	Exp.		Obs.	Exp.		Obs.	Exp.	
Between different types for all ages of stands												
RP	QV	7	12	−7.19	0.05	0.024	−1.33	0.48	0.397	−7.19	0.05	0.024
RP	PO	7	12	2.14	0.33	0.288	11.00	0.14	0.049	2.14	0.33	0.288
QV	PO	12	12	9.33	0.00	0.004	12.33	0.33	0.021	9.33	0.00	0.004
Between different age groups for *R. pseudoacacia* stands												
AG1	AG2	2	3	−7.33	−4.00	0.100	−7.33	−0.67	0.097	1.33	1.33	0.513
AG1	AG3	2	2	4.00	−4.00	0.494	0.00	0.00	0.492	8.00	−8.00	0.499
AG2	AG3	3	2	11.33	−2.00	0.407	7.33	−2.67	0.298	6.67	0.00	0.298
Between different age groups for *Q. variabilis* stands												
AG1	AG2	4	4	−1.00	1.00	0.322	−8.00	0.00	0.156	−5.00	1.00	0.143
AG1	AG3	4	4	21.00	−1.00	0.038	2.00	0.00	0.428	−11.00	1.00	0.013
AG2	AG3	4	4	22.00	0.00	0.036	10.00	0.00	0.194	−6.00	0.00	0.112
Between different age groups for *P. orientalis* stands												
AG1	AG2	6	3	−6.67	1.33	0.037	−12.67	−0.67	0.075	−6.67	1.33	0.037
AG1	AG3	6	3	0.00	0.00	0.755	−4.67	−0.67	0.202	0.00	0.00	0.755
AG2	AG3	3	3	6.67	4.00	0.346	8.00	0.00	0.271	6.67	4.00	0.346

G_1: Group 1; G_2: Group 2; n1 and n2: number of plots for Group 1 and Group 2 respectively; AG: age group; RP: *R. pseudoacacia*; QV: *Q. variabilis*; PO: *P. orientalis*; p_v: p_value.

If component regenerating species were considered, both the recruits density and frequency showed similar patterns (Table 1 and Table 3). For the oak stands, the planted species (i.e. the oak) had both higher density and frequency than the other species not planted in the focal stands. But for the other two types of stands, generally the planted species (i.e. the black locust and the arborvitae respectively) had lower densities and frequencies than other species not planted in the focal stands. Black locust had established recruits in the oak and arborvitae stands, and oak had established recruits in the black locust and arborvitae stands. But there were no arborvitae recruits in the oak and black locust stands. At least five other broadleaved deciduous species had recruits in all the three types of stands, which include *Ailanthus altissima, Pistacia chinensis, Ulmus spp., Melia azedarach* and *Broussonetia papyrifera*.

### Correlations of recruits density with forest attributes

Stand age was only significantly correlated with the density of small saplings (p = 0.009) and marginally correlated with the density of seedlings (p = 0.089) for arborvitae stands, and significantly correlated with the density of large saplings for oak stands (p = 0.01), which were all positive (Table 5). But it was not correlated with the density of the recruits in the other situations. When correlations between the number of seedlings and the other stand attributes were considered, a consistent pattern appeared among the three types of stands. That is, the density of seedlings was positively correlated with the overstory cover and negatively correlated with the covers of shrub, herb and litter, most of which were statistically significant. Generally stand age was at least not strongly correlated with the density of seedlings (p≥0.089).

**Table 5:** Correlation analysis between recruits density and stand attributes using Kendall's correlation coefficient and randomization test.

	Seedling		Small sapling		Large sapling	
Stand attribute	Correlation	p-value	Correlation	p-value	Correlation	p-value
*R. pseudoacacia* stands						
Stand age	−0.108	0.322	0.150	0.289	−0.296	0.198
Overstory cover	0.685	0.009	−0.683	0.009	0.000	0.438
Shrub cover	−0.617	0.015	0.524	0.035	0.282	0.157
Herb cover	−0.309	0.141	0.238	0.205	0.169	0.242
Litter cover	−0.411	0.070	0.333	0.103	0.507	0.058
*Q. variabilis* stands						
Stand age	−0.208	0.180	−0.070	0.371	0.569	0.010
Overstory cover	0.299	0.078	−0.222	0.161	0.097	0.332
Shrub cover	−0.469	0.013	0.110	0.328	0.240	0.160
Herb cover	−0.079	0.325	−0.095	0.349	−0.226	0.144
Litter cover	−0.554	0.001	−0.016	0.406	0.173	0.216
*P. orientalis* stands						
Stand age	0.331	0.089	0.505	0.009	0.297	0.131
Overstory cover	0.281	0.086	0.400	0.031	0.430	0.034
Shrub cover	−0.144	0.259	−0.158	0.239	−0.249	0.165
Herb cover	−0.469	0.015	−0.215	0.171	0.019	0.462
Litter cover	−0.543	0.005	−0.382	0.039	−0.111	0.306

However, there was no consistent pattern for the correlation of the density of small saplings with the stand attributes (Table 5). Stand age was only significantly and positively correlated with the density of small saplings for arborvitae stands (p = 0.009) and with the density of large saplings for oak stands (p = 0.01). The correlations between overstory cover and the density of small saplings and that of large saplings were significantly positive for arborvitae stands (p = 0.031 and 0.034), but negative for black locust stands (p = 0.009). While litter cover was negatively correlated with the small saplings for arborvitae stands (p = 0.039), it was positively correlated with the large saplings for black locust stands (p = 0.058).

## Discussions

Forest regeneration is a complex process, in which many factors are involved [20]. In this study we have only considered some forest attributes, including overstory cover, shrub cover, herb cover and litter cover as well as stand age. We have found that for all the three types of forests the density of seedlings was negatively correlated with litter cover, which is consistent with previous studies, e.g. Facelli and Pickett [21] and Hérault et al. [22]. This means that the litter layer in the forests is a barrier to the regeneration of trees. Although the litter layer can prevent seedlings from being damaged by the freezing temperature in winter, which is helpful for tree regeneration [23], the litter layer acts as a barrier so that the seeds are difficult to touch the soil which consequently affects tree regeneration [1], [24]. Usually this barrier effect increases with the thickness of the litter layer [23]. Although we only recorded the cover of the litter layer and did not measure its thickness, we can reasonably assume that cover and thickness of the litter layer are generally positively correlated. Probably the cover of litter layer enhances this barrier effect. From this reasoning it can be easily explained why the arborvitae forests have fewer tree seedlings than both the black locust forests and the oak forests given the higher litter cover (43.83%) in the arborvitae forests compared to 34.44% in the black locust forests and 12.08% in the oak forests. In addition, it has been demonstrated that seedling emergence of large-seeded species may not be strongly limited by the litter thickness [22], [25], [26], which explains why there are more oak seedlings.

We have also found that the density of seedlings was negatively correlated with shrub cover and herb cover. This means that shrubs and herbs compete with seedlings, and their ecological niches may have some overlap. This is in line with Bloor et al. [27], Laliberté et al. [28], Liira et al. [4], Harmer et al. [29], Luo and Zheng [23] and Wu et al. [30]. Harmer et al. [29] pointed out that survival and growth of ash seedlings can be adversely affected by the ground flora, which can cause significant reductions in height due to root competition. Bailey et al. [31] found that seedling safe sites are related to low cover of grass. Bloor et al. [27] observed that tree seedlings compete with grass for soil resources but not for light.

The light reaching the ground layer is largely determined by the overstory canopy, and it has been found to be determinant for regeneration dynamics [22], [32]. A lack of light generally decreases not only the survival [33]–[35], but also the establishment and growth of tree seedlings [22], [29], [30], [32], [36]–[40]. Bailey et al. [31] also observed that seedling safe sites are related to canopy gaps. However, Zhu et al. [41] observed that seedling density was not correlated with stand openness. In our study, seedling density is positively correlated with the overstory canopy cover, which is contrary to most of the previous findings. Although all the three species in our study are light-loving plants, their juveniles can tolerate shade to some degree. Therefore, the canopy cover may not severely affect the seedling density in a negative way. The main reason for the positive correlation between seedling density and overstory canopy cover may be through the suppression of understorey cover (including both shrubs and herbs) by the canopy cover, which leaves space, water and nutrients for tree seedlings. It has been demonstrated that shading is essential for limiting the occurrence of tall persistent and strongly competitive herbs and finally for regeneration of trees [22], [32]. Another possible reason is through the adjustment of the humidity and temperature within forests, especially in the upper layer of soil, by the overstory canopy. Although the annual precipitation is above 600 mm in the study area, the uneven temporal distribution of the precipitation makes frequent occurrence of drought in spring. The higher cover of the overstory canopy increases the humidity in the top layer of soil, which prevents the seedlings from suffering drought in the dry season. This is supported by Sánchez-Gómez et al. [42] who found that the impact of drought on the survival and relative growth rate of seedlings was stronger in high light than in deep shade. Furthermore, the higher humidity facilitates the decomposition of the litters, which in turn reduces the barrier effect of the litter layer and therefore helps seedlings to establish. Additionally, the lowest temperature in winter in the study area can drop to −20°C, and the higher cover of the overstory canopy increases the lowest temperature within forests in winter, which helps the seedlings to avoid being damaged by the extreme temperature. All these facilitate the establishment and growth of seedlings. This finding suggests that overstory canopy should be protected at least up to this stage of the restoration, any reduction of canopy cover may reduce the density of seedlings and therefore affect the regeneration.

In this study we have found that the black locust stands and the oak stands had around 2000 seedlings and more than 1000 saplings ha^−1^. Both fall into the middle category according to the criteria for natural regeneration [23]. However, while the arborvitae stands had more than 1000 saplings ha^−1^, they only had little more than 700 seedlings ha^−1^ which falls into the low category according to the criteria for natural regeneration [23]. These are consistent with the findings of Luo and Zheng [23] for the arborvitae forests in a mountainous area in the Beijing region. Furthermore, the recruits had low frequencies and were not evenly distributed. All these indicate that difficulty exists in the regeneration of trees in the arborvitae stands. Further silvicultural measures may be needed. At least human disturbance should be avoided.

We have also found that many stands had established seedlings and even saplings of some tree species other than the planted ones, which are all native broadleaved deciduous species. This means that these tree species have high dispersal ability and enough source of seeds in this area. In addition, multi-species may make fuller use of the safe regeneration microsites since the seedlings of different species may have different environmental requirements (even at the micro scale) and therefore they usually occupy different sites. This suggests that planting multiple species in the same area at the beginning of afforestation may be better than single species in terms of regeneration, which helps to maintain a higher level of biodiversity.

In summary, while our findings are consistent with previous studies on the negative relationships between seedling density and the coverage of shrub, herb and litter layers, we do have found a positive relationship between seedling density and upperstory coverage, which is contradictory to most previous studies. This means that in our study area, higher upperstory cover leads to better forest regeneration. It has practical implications for forest management in this area. That is, in order to facilitate forest regeneration, the upperstory coverage should be maintained at a high level, and any destruction to the upperstory trees should be avoided; if possible, it is even better to protect the forests in this area.

## Supporting Information

Table S1 **Forest types, locations, stand attributes, and numbers and frequencies of seedlings and saplings for the 33 plots used in the study.**
(DOCX)
