# Supplementary material for: Natural Regeneration of Trees in Three Types of Afforested Stands in the Taihang Mountains, China
Source: PLoS One. 2014 Sep 30;9(9):e108744. doi: 10.1371/journal.pone.0108744 (PMC4182556; doi:10.1371/journal.pone.0108744)
Supplement: Table S1 — Forest types, locations, stand attributes, and numbers and frequencies of seedlings and saplings for the 33 plots used in the study. (DOCX) [file pone.0108744.s001.docx]

**Table S1** Forest type, stand attributes, number and frequency of seedlings and saplings for the 33 plots used in this study.

| Plot | FT | Easting | Northing | SA | OC | SC | HC | LC | NSD | NSS | NLS | FSD | FSS | FLS |
| --- | --- | --- | --- | --- | --- | --- | --- | --- | --- | --- | --- | --- | --- | --- |
| 1 | RP | 614209 | 3888261 | 40 | 70 | 14 | 25 | 28 | 10 | 8 | 2 | 0.4 | 0.28 | 0.08 |
| 2 | RP | 614121 | 3888306 | 40 | 60 | 35 | 21 | 40 | 5 | 10 | 3 | 0.2 | 0.32 | 0.12 |
| 3 | PO | 616646 | 3888678 | 20 | 20 | 45 | 20 | 57 | 2 | 4 | 1 | 0.08 | 0.12 | 0.04 |
| 4 | PO | 616319 | 3888710 | 30 | 70 | 25 | 8 | 26 | 15 | 7 | 1 | 0.32 | 0.2 | 0.04 |
| 5 | PO | 615803 | 3889751 | 50 | 45 | 67 | 5 | 35 | 13 | 17 | 0 | 0.2 | 0.4 | 0 |
| 6 | QV | 616108 | 3889564 | 55 | 75 | 10 | 2 | 10 | 2 | 28 | 8 | 0.04 | 0.48 | 0.28 |
| 7 | QV | 616279 | 3889123 | 30 | 75 | 12 | 8 | 13 | 7 | 23 | 5 | 0.2 | 0.44 | 0.12 |
| 8 | RP | 614732 | 3882603 | 20 | 30 | 55 | 23 | 65 | 5 | 16 | 0 | 0.16 | 0.28 | 0 |
| 9 | PO | 615834 | 3877834 | 20 | 20 | 13 | 12 | 20 | 10 | 11 | 0 | 0.28 | 0.32 | 0 |
| 10 | PO | 620760 | 3877107 | 20 | 40 | 8.5 | 37 | 41 | 5 | 20 | 2 | 0.16 | 0.4 | 0.04 |
| 11 | PO | 622523 | 3879766 | 10 | 5 | 45 | 66 | 74 | 1 | 5 | 0 | 0.04 | 0.16 | 0 |
| 12 | QV | 622734 | 3884935 | 40 | 75 | 18 | 5 | 20 | 23 | 9 | 3 | 0.4 | 0.32 | 0.12 |
| 13 | QV | 622727 | 3885004 | 40 | 76 | 5 | 8 | 6 | 68 | 32 | 2 | 0.4 | 0.36 | 0.04 |
| 14 | RP | 616289 | 3888963 | 20 | 63 | 34 | 17 | 46 | 13 | 2 | 5 | 0.36 | 0.08 | 0.16 |
| 15 | PO | 635687 | 3879014 | 10 | 10 | 75 | 36 | 90 | 0 | 0 | 0 | 0 | 0 | 0 |
| 16 | PO | 634624 | 3879156 | 52 | 85 | 45 | 6 | 47 | 6 | 5 | 0 | 0.2 | 0.2 | 0 |
| 17 | PO | 634638 | 3879186 | 52 | 83 | 8.5 | 14 | 9 | 9 | 22 | 4 | 0.12 | 0.28 | 0.04 |
| 18 | RP | 635021 | 3877406 | 55 | 40 | 24 | 13 | 26 | 5 | 12 | 0 | 0.04 | 0.2 | 0 |
| 19 | QV | 633202 | 3880368 | 15 | 40 | 30 | 12 | 25 | 21 | 12 | 3 | 0.44 | 0.24 | 0.08 |
| 20 | QV | 632946 | 3879927 | 15 | 73 | 16 | 4 | 15 | 8 | 19 | 4 | 0.24 | 0.44 | 0.16 |
| 21 | PO | 634651 | 3879608 | 30 | 80 | 48 | 19 | 50 | 15 | 9 | 0 | 0.36 | 0.28 | 0 |
| 22 | QV | 634183 | 3878885 | 55 | 70 | 5 | 13 | 10 | 10 | 4 | 3 | 0.24 | 0.12 | 0.08 |
| 23 | QV | 634217 | 3878918 | 55 | 85 | 20 | 5 | 22 | 1 | 4 | 10 | 0.04 | 0.16 | 0.2 |
| 24 | RP | 612070 | 3884612 | 20 | 90 | 55 | 32 | 50 | 173 | 8 | 6 | 0.48 | 0.2 | 0.16 |
| 25 | PO | 615977 | 3891283 | 35 | 89 | 46 | 13 | 52 | 2 | 13 | 6 | 0.08 | 0.2 | 0.2 |
| 26 | QV | 616057 | 3889934 | 40 | 89 | 5 | 2 | 5 | 30 | 8 | 6 | 0.48 | 0.24 | 0.2 |
| 27 | RP | 616330 | 3888501 | 30 | 70 | 12 | 11 | 15 | 64 | 7 | 0 | 0.4 | 0.16 | 0 |
| 28 | QV | 616327 | 3888490 | 55 | 80 | 7 | 3 | 7 | 29 | 10 | 8 | 0.28 | 0.2 | 0.16 |
| 29 | RP | 613920 | 3888906 | 50 | 86 | 6 | 18 | 10 | 35 | 6 | 0 | 0.4 | 0.16 | 0 |
| 30 | QV | 623042 | 3886424 | 15 | 76 | 6 | 4 | 8 | 23 | 8 | 0 | 0.4 | 0.2 | 0 |
| 31 | QV | 621984 | 3887306 | 15 | 90 | 4 | 4.5 | 4 | 41 | 6 | 1 | 0.36 | 0.16 | 0.04 |
| 32 | RP | 624121 | 3871211 | 35 | 40 | 17 | 18 | 30 | 214 | 9 | 2 | 0.4 | 0.28 | 0.08 |
| 33 | PO | 622946 | 3871052 | 10 | 15 | 8 | 20 | 25 | 9 | 2 | 0 | 0.2 | 0.08 | 0 |

FT: forest type; SA: stand age; OC: overstory cover; SC: shrub cover; HC: herb cover; LC: litter cover;

NSD: number of seedlings; NSS: number of small saplings; NLS: number of large saplings;

FSD: frequency of seedlings; FSS: frequency of small saplings; FLS: frequency of large saplings; RP: *R.* *pseudoacacia*; QV: *Q.* *variabilis*; PO: *P.* *orientalis*.
